# Supplementary material for: Invariant point message passing for protein side chain packing
Source: bioRxiv. 2023 Dec 21:2023.08.03.551328. Preprint. [Version 2] doi: 10.1101/2023.08.03.551328 (PMC10769188; doi:10.1101/2023.08.03.551328)
Supplement: Supplement 1 [file media-1.pdf]

Supplemental Material

TITLE:

Invariant point message passing for protein side chain packing

RUNNING TITLE:

Protein side chain packing

Nicholas Z. Randolph[1,2] and Brian Kuhlman[1,2]

[1]Department of Bioinformatics and Computational Biology, University of North Carolina School of Medicine, Chapel Hill, North Carolina, USA.

[2]Department of Biochemistry and Biophysics, University of North Carolina School of Medicine, Chapel Hill, North Carolina, USA.

Corresponding Author: B.K. - bkuhlman@email.unc.edu

Table of Contents **Page**

Table of Contents	Page
Figure S1: PIPPack performance across recycles .	3
Figure S2: Representative Clashes Produced by PIPPack.	4
Table S1: Side chain χ angle errors across amino acid type.	5
Table S2: Rotamer Evaluations for PSCP Methods.	10

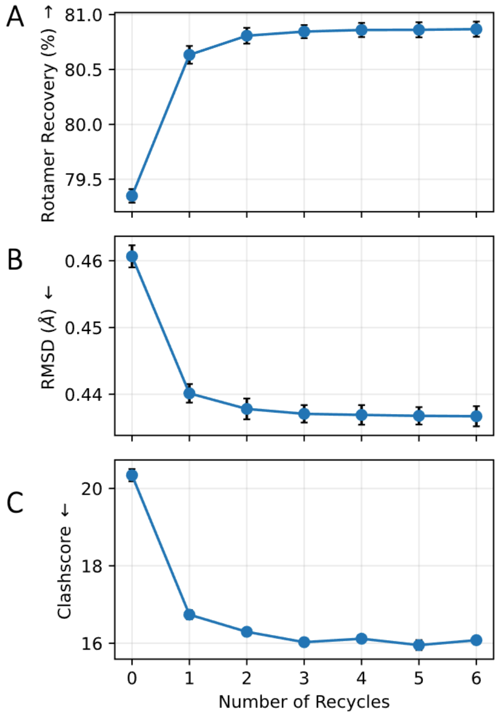

Figure S1: PIPPack performance across recycles. PIPPack uses recycling to iteratively refine its side chain conformation predictions, resulting in improved performance metrics. Recycling past the value PIPPack was trained for (in our case 3) does not result in any significant differences in performance, but the model performs well even with a single recycle.

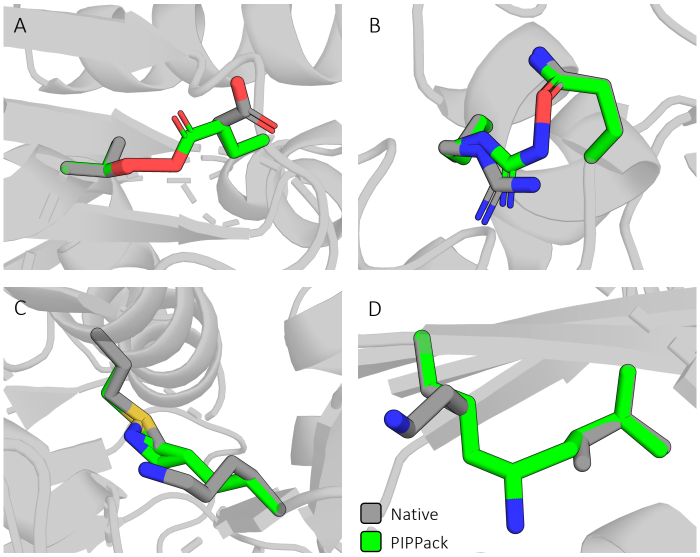

Figure S2: Representative Clashes Produced by PIPPack. Although not common, PIPPack can produce atomic clashes due to the one-shot nature of the network. Egregious clashes, as shown here, are usually fixed via post-prediction minimization. Most of these clashes occur between hydrogen bonding partners (A and B) and/or long side chains (B, C, and D).

Table S1: Side chain χ angle errors across amino acid type.

AA Type	Method	MAE (°) ↓				AA Type	Method	MAE (°) ↓	
		χ_1	χ_2	χ_3	χ_4			χ_1	χ_2
ARG	Rosetta Packer	23.00	24.34	48.62	48.54	ASN	Rosetta Packer	20.35	28.72
	DLPacker	17.11	22.76	45.97	53.64		DLPacker	13.19	28.56
	AttnPacker	15.61	20.11	43.56	48.96		AttnPacker	12.07	27.69
	AttnPacker+PP	17.22	21.08	42.78	48.56		AttnPacker+PP	12.36	53.56
	DiffPack	16.50	19.86	41.19	46.56		DiffPack	14.29	33.27
	DiffPack +Confidence	<u>13.30</u>	<u>17.18</u>	<u>34.86</u>	**40.30**		DiffPack +Confidence	11.74	36.16
	PIPPack†	13.60	17.79	36.15	42.36		PIPPack†	11.24	23.66
	PIPPack+RS†	13.90	18.03	36.67	42.34		PIPPack+RS†	11.24	24.42
	PIPPack (ensembled)	**12.62**	**16.46**	**33.98**	<u>39.79</u>		PIPPack (ensembled)	**10.22**	**22.06**
	PIPPack+RS (ensembled)	13.54	17.61	35.97	41.68		PIPPack+RS (ensembled)	<u>10.66</u>	<u>23.17</u>
LYS	Rosetta Packer	23.40	25.05	32.14	41.89	ASP	Rosetta Packer	19.72	18.1
	DLPacker	17.03	24.37	38.45	60.48		DLPacker	12.86	15.40
	AttnPacker	14.77	20.50	30.16	41.27		AttnPacker	10.40	14.09
	AttnPacker+PP	15.64	21.08	30.50	41.37		AttnPacker+PP	10.80	13.96
	DiffPack	15.73	19.67	28.48	40.06		DiffPack	11.95	12.67
	DiffPack +Confidence	<u>13.35</u>	**17.83**	**25.73**	<u>38.45</u>		DiffPack +Confidence	**9.59**	<u>11.55</u>
	PIPPack†	14.09	19.22	26.46	38.09		PIPPack†	10.44	11.92
	PIPPack+RS†	14.20	19.50	26.85	39.28		PIPPack+RS†	10.55	12.34
	PIPPack (ensembled)	**13.12**	<u>18.23</u>	<u>25.76</u>	**36.99**		PIPPack (ensembled)	**9.59**	**11.28**
	PIPPack+RS (ensembled)	13.59	18.84	26.41	38.71		PIPPack+RS (ensembled)	<u>10.03</u>	11.95
GLU	Rosetta Packer	29.33	33.46	27.00		LEU	Rosetta Packer	12.64	18.53

	DLPacker	23.15	29.94	30.35			DLPacker	8.33	14.25
	AttnPacker	18.82	24.44	26.82			AttnPacker	7.13	87.63
	AttnPacker+PP	19.34	24.57	26.36			AttnPacker+PP	7.36	13.44
	DiffPack	19.55	25.16	22.54			DiffPack	9.57	11.77
	DiffPack +Confidence	16.35	21.31	20.48			DiffPack +Confidence	7.33	9.49
	PIPPack†	17.48	22.94	21.49			PIPPack†	6.18	9.71
	PIPPack+RS†	17.79	23.33	22.06			PIPPack+RS†	6.19	9.76
	PIPPack (ensembled)	**16.33**	**21.24**	**20.41**			PIPPack (ensembled)	**5.70**	**9.00**
	PIPPack+RS (ensembled)	16.86	22.27	21.41			PIPPack+RS (ensembled)	5.77	9.18
**GLN**	Rosetta Packer	23.20	32.63	42.28		**ILE**	Rosetta Packer	12.48	19.03
	DLPacker	18.17	27.87	43.74			DLPacker	6.86	17.78
	AttnPacker	15.67	24.47	42.01			AttnPacker	6.06	14.92
	AttnPacker+PP	16.29	24.68	71.75			AttnPacker+PP	6.43	15.07
	DiffPack	16.95	24.76	46.42			DiffPack	4.66	11.52
	DiffPack +Confidence	13.99	21.43	46.27			DiffPack +Confidence	**4.02**	**9.64**
	PIPPack†	14.25	22.07	39.36			PIPPack†	5.01	10.93
	PIPPack+RS†	14.24	22.13	40.14			PIPPack+RS†	5.03	11.02
	PIPPack (ensembled)	**13.23**	**20.35**	**37.88**			PIPPack (ensembled)	4.63	10.07
	PIPPack+RS (ensembled)	13.54	21.16	38.80			PIPPack+RS (ensembled)	4.71	10.34
**MET**	Rosetta Packer	17.77	21.39	39.55		**PRO**	Rosetta Packer	10.90	15.14
	DLPacker	13.61	20.02	41.74			DLPacker	8.45	12.42
	AttnPacker	12.16	17.52	43.65			AttnPacker	8.10	11.74
	AttnPacker+PP	13.02	17.90	43.06			AttnPacker+PP	8.43	12.28
	DiffPack	15.53	20.05	40.67			DiffPack	5.28	6.35

	DiffPack +Confidence	11.55	15.30	32.51			DiffPack +Confidence	**5.17**	**6.20**
	PIPPack†	10.25	13.40	30.90			PIPPack†	6.97	9.24
	PIPPack+RS†	10.33	13.59	30.44			PIPPack+RS†	7.52	10.18
	PIPPack (ensembled)	**9.46**	**11.95**	**28.66**			PIPPack (ensembled)	6.56	8.62
	PIPPack+RS (ensembled)	9.69	12.72	28.76			PIPPack+RS (ensembled)	7.28	9.89
**PHE**	Rosetta Packer	13.14	11.70			**THR**	Rosetta Packer	14.46	
	DLPacker	6.45	8.85				DLPacker	10.05	
	AttnPacker	5.26	8.56				AttnPacker	8.15	
	AttnPacker+PP	5.81	8.59				AttnPacker+PP	8.44	
	DiffPack	8.32	7.56				DiffPack	7.78	
	DiffPack +Confidence	6.49	6.50				DiffPack +Confidence	6.91	
	PIPPack†	5.12	**5.99**				PIPPack†	7.31	
	PIPPack+RS†	5.12	6.07				PIPPack+RS†	7.50	
	PIPPack (ensembled)	**4.71**	8.00				PIPPack (ensembled)	**6.61**	
	PIPPack+RS (ensembled)	4.84	7.75				PIPPack+RS (ensembled)	6.93	
**TYR**	Rosetta Packer	14.98	11.63			**SER**	Rosetta Packer	32.99	
	DLPacker	7.06	8.82				DLPacker	22.08	
	AttnPacker	5.99	8.92				AttnPacker	18.20	
	AttnPacker+PP	6.39	8.96				AttnPacker+PP	18.46	
	DiffPack	8.99	7.47				DiffPack	21.53	
	DiffPack +Confidence	7.32	6.62				DiffPack +Confidence	20.17	
	PIPPack†	5.81	**6.23**				PIPPack†	17.03	
	PIPPack+RS†	5.92	6.45				PIPPack+RS†	17.51	

	PIPPack (ensembled)	**5.29**	8.13				PIPPack (ensembled)	**15.19**	
	PIPPack+RS (ensembled)	5.60	7.71				PIPPack+RS (ensembled)	16.33	
	Rosetta Packer	17.63	30.62				Rosetta Packer	11.73	
	DLPacker	6.99	14.97				DLPacker	10.48	
	AttnPacker	6.84	15.76				AttnPacker	6.83	
	AttnPacker+PP	7.11	15.39				AttnPacker+PP	8.58	
	DiffPack	10.53	26.73				DiffPack	8.27	
**TRP**	DiffPack +Confidence	8.30	14.77			**CYS**	DiffPack +Confidence	6.14	
	PIPPack†	6.25	12.13				PIPPack†	5.94	
	PIPPack+RS†	6.33	12.07				PIPPack+RS†	6.02	
	PIPPack (ensembled)	**5.51**	**10.57**				PIPPack (ensembled)	**5.23**	
	PIPPack+RS (ensembled)	5.95	10.79				PIPPack+RS (ensembled)	5.75	
	Rosetta Packer	18.61	45.62				Rosetta Packer	14.02	
	DLPacker	10.48	47.03				DLPacker	7.54	
	AttnPacker	9.54	52.95				AttnPacker	32.93	
	AttnPacker+PP	10.32	69.95				AttnPacker+PP	7.18	
	DiffPack	12.77	53.06				DiffPack	5.21	
**HIS**	DiffPack +Confidence	9.74	49.34			**VAL**	DiffPack +Confidence	**4.62**	
	PIPPack†	8.95	37.93				PIPPack†	5.83	
	PIPPack+RS†	8.98	38.17				PIPPack+RS†	5.85	
	PIPPack (ensembled)	**8.05**	**34.37**				PIPPack (ensembled)	5.47	

	PIPPack+RS (ensembled)	8.51	35.90				PIPPack+RS (ensembled)	5.56	

Table S2: Rotamer Evaluations for PSCP Methods.

Method	Rotamer Evaluation (%)		
	**Favored**	**Allowed**	**Outlier**
Native	96.77	2.54	0.69
Rosetta Packer	99.29	0.65	0.07
DLPacker	96.33	2.50	1.17
AttnPacker	87.65	8.34	4.01
AttnPacker+PP	93.62	4.09	2.29
DiffPack	97.97	1.48	0.55
DiffPack +Confidence	98.35	1.23	0.43
PIPPack	98.93	0.90	0.17
PIPPack+RS	98.71	1.08	0.21
PIPPack (ensembled)	99.03	0.82	0.14
PIPPack+RS (ensembled)	98.78	1.03	0.19
